# Supplementary material for: Periodontitis induces skeletal muscle atrophy by increasing circulating levels of activin A
Source: Nat Commun. 2026 May 6;17:4063. doi: 10.1038/s41467-026-72766-1 (PMC13149552; doi:10.1038/s41467-026-72766-1)

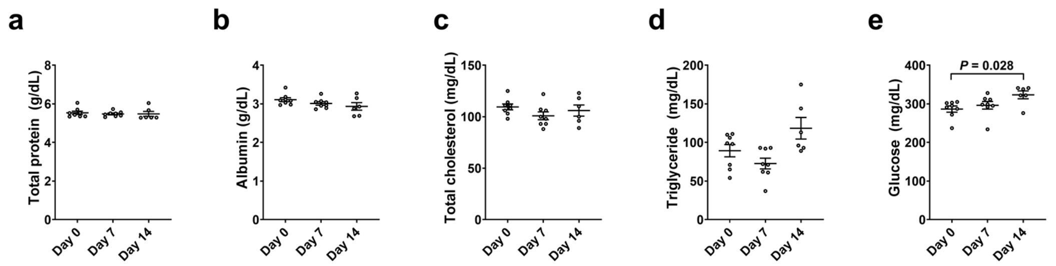

Supplementary Fig. 1: Postprandial blood nutrient analysis in the ligature-induced periodontitis (LIP) model.

a-e Changes in postprandial levels of total protein (**a**), albumin (**b**), total cholesterol (**c**), triglyceride (**d**), and glucose (**e**) after ligature placement. (Day 0 and 7: n = 8, Day 14: n = 6). These nutrient levels remained stable or elevated following ligature placement, indicating that the LIP model did not impair nutrient absorption.

Data represent mean ± SEM. Statistical significance was assessed by one-way ANOVA with Holm-Šídák post hoc test. Means of each time point were compared against the Day 0 group. Source data are provided as a Source Data file.

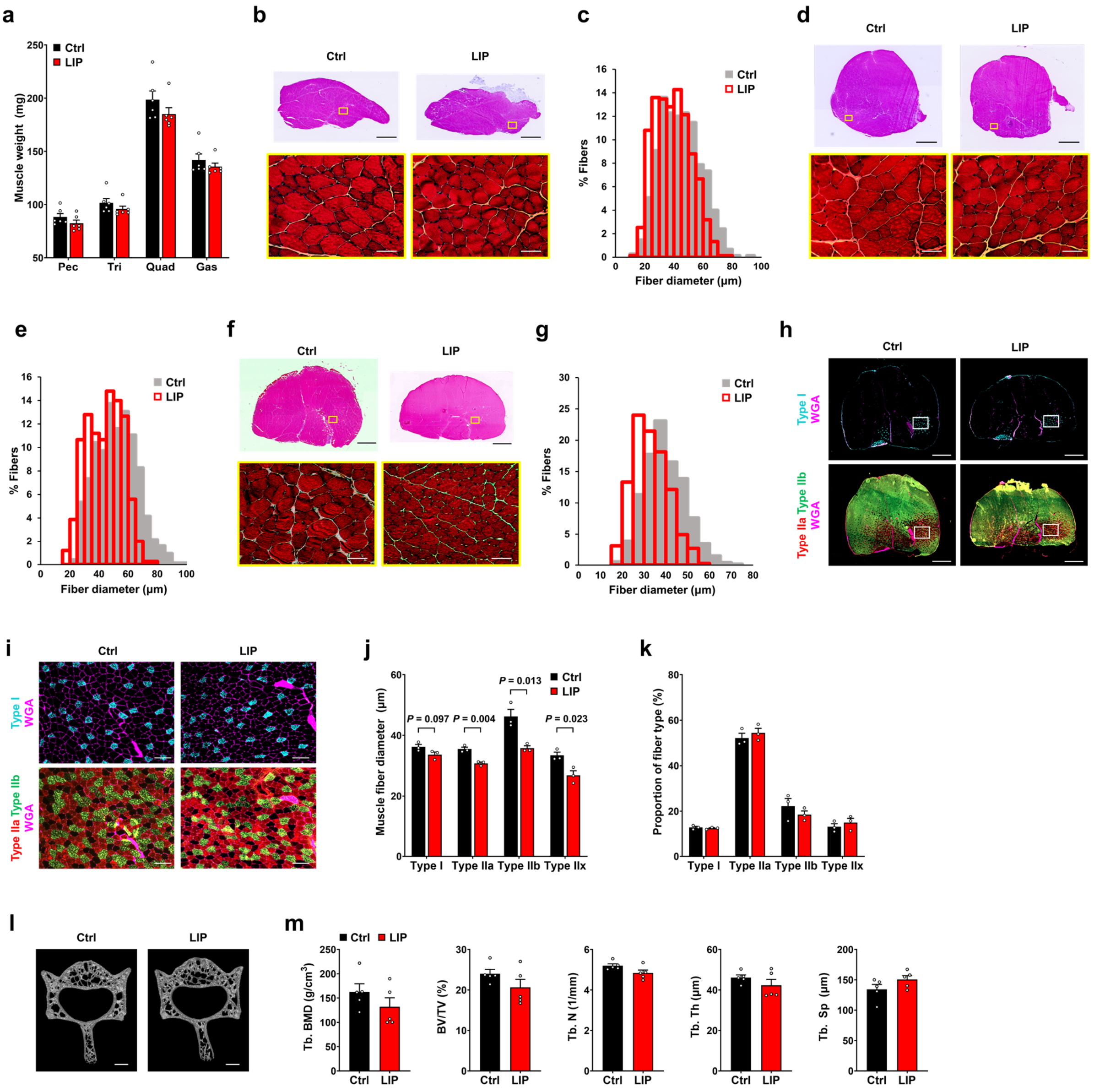

Supplementary Fig. 2: Muscle histology and lumbar histomorphometric analysis in the LIP model.

a Weights of the pectoralis (Pec), triceps (Tri), quadriceps (Quad), and gastrocnemius (Gas) muscles in control and periodontitis-induced mice 7 days after ligature placement (n = 6 each).

b, d, f H&E-stained sections of pectoralis (**b**), triceps (**d**), and gastrocnemius (**f**) muscles 14 days after ligature placement. Magnified images of the yellow-boxed regions are shown in the lower panels. Scale bars: 1 mm (top), 50 μm (bottom).

c, e, g Distribution of muscle fiber diameters in the pectoralis (**c**), triceps (**e**), and gastrocnemius (**g**) muscles (n = 3 each). Fiber diameters were plotted as a percentage of the total fiber number (750 fibers per group). Mean fiber diameters (Ctrl vs. LIP): 44.15 ± 0.50 vs. 39.21 ± 0.44 μm (**c**), 51.70 ± 0.51 vs. 43.84 ± 0.44 μm (**e**), and 39.32 ± 0.34 vs. 32.96 ± 0.29 μm (**g**).

h, i Representative fiber-type-stained images of gastrocnemius muscles (**h**) and magnified views of the

white-boxed regions (**i**) 14 days after ligature placement. For fiber-type analysis, an 800 μm x 600 μm area (white-boxed region) was selected to capture maximum diversity of fiber types. Scale bars: 1 mm (**h**), 100 μm (**i**).

j Muscle fiber diameter by fiber type shown in (**i**) (*n* = 3 each). Type **II** fibers showed significant diameter reduction, while type **I** fibers also showed a mild reduction.

k Proportion of fiber types shown in (**i**) (*n* = 3 each). Periodontitis-induced mice exhibited a slight fiber-type shift from type **II**b toward type **II**a and **II**x fibers.

l Representative micro-CT images of the lumbar spine in control and periodontitis-induced mice 14 days after ligature placement. Scale bar, 0.5 mm.

m Although not statistically significant, reductions were observed in trabecular bone mineral density (Tb. BMD), bone volume/total volume of interest (BV/TV), and trabecular bone number (Tb. N) in the LIP group (*n* = 5 each).

Data represent mean ± SEM. Statistical significance was assessed by two-tailed Student's *t*-test (**a, j, k, m**). Source data are provided as a Source Data file.

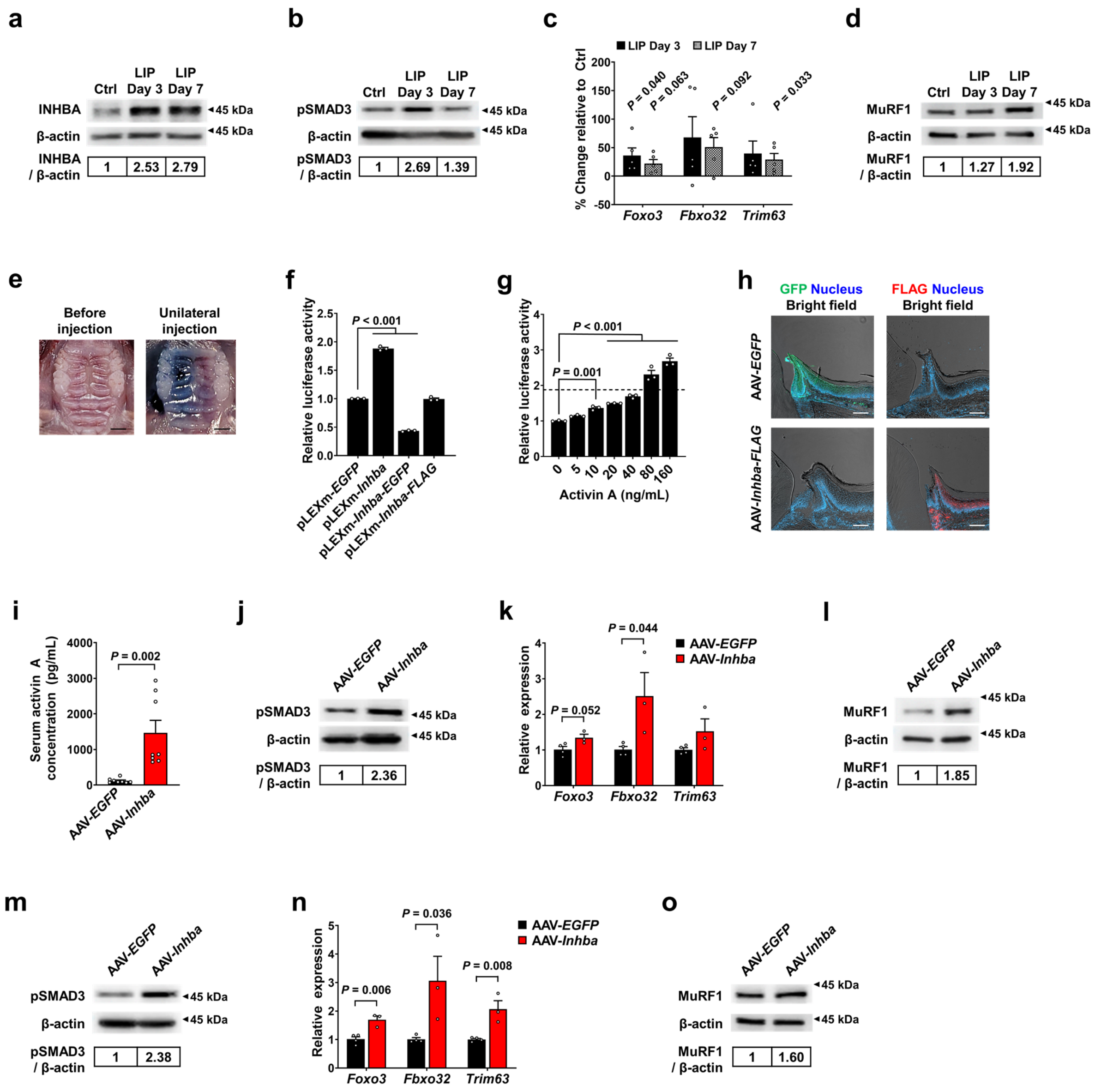

Supplementary Fig. 3: Activation of skeletal muscle activin signaling by gingiva-derived activin A.

a Representative Western blot of INHBA protein in gingival tissue from control and periodontitis-induced mice. INHBA protein levels were elevated in periodontitis-affected gingiva compared with controls, confirming upregulation of INHBA expression (~45 kDa) at the protein level.

b Representative Western blot of phosphorylated SMAD3 (pSMAD3) in extensor digitorum longus (EDL) muscles.

c Percent change in expression of muscle atrophy-related genes (*Foxo3*, *Fbxo32*, and *Trim63*) in EDL muscles of periodontitis-affected mice relative to controls (*n* = 5 each).

d Representative Western blot of MuRF1 (encoded by *Trim63*) in EDL muscles of periodontitis-induced mice compared with controls.

e Representative image of unilateral intra-gingival Evans blue dye injection.

f, g Luciferase reporter assays measuring SMAD signaling activation in C3H10T1/2 cells (*n* = 3 each).

SMAD activation induced by untagged *Inhba* was comparable to treatment with 40–80 ng/mL recombinant activin A (**g**) (*n* = 3 each).

h Immunofluorescence images showing GFP (green) and FLAG (red) expression in gingival tissue from mice injected with AAV-*EGFP* or AAV-*Inhba-FLAG*. Scale bar, 100 μm.

i Serum activin A levels in mice transduced with AAV-*EGFP* or AAV-*Inhba* in gingival tissue (*n* = 8 each).

j, m Representative Western blots of pSMAD3 in quadriceps (**j**) and EDL (**m**) muscles from mice injected with AAV-*EGFP* or AAV-*Inhba*.

k, n qRT-PCR analysis of muscle atrophy-related genes (*Foxo3*, *Fbxo32*, and *Trim63*) in quadriceps (**k**) and EDL (**n**) muscles from mice injected with AAV-*EGFP* or AAV-*Inhba* (AAV-*EGFP*: *n* = 4, AAV-*Inhba*: *n* = 3).

l, o Representative Western blots of MuRF1 in quadriceps (**l**) and EDL (**o**) muscles from mice injected with AAV-*EGFP* or AAV-*Inhba*.

Data (**c, f, g, i, k, n**) represent mean ± SEM. Statistical significance was assessed by two-tailed Student's *t*-test (**c, i, k, n**), or one-way ANOVA with Holm-Šídák post hoc test (**f, g**). In (**f**), means were compared between the pLEXm-*EGFP* group and other groups; in (**g**), means were compared between the non-treated group and other groups. Source data and exact *P* values are provided as a Source Data file.

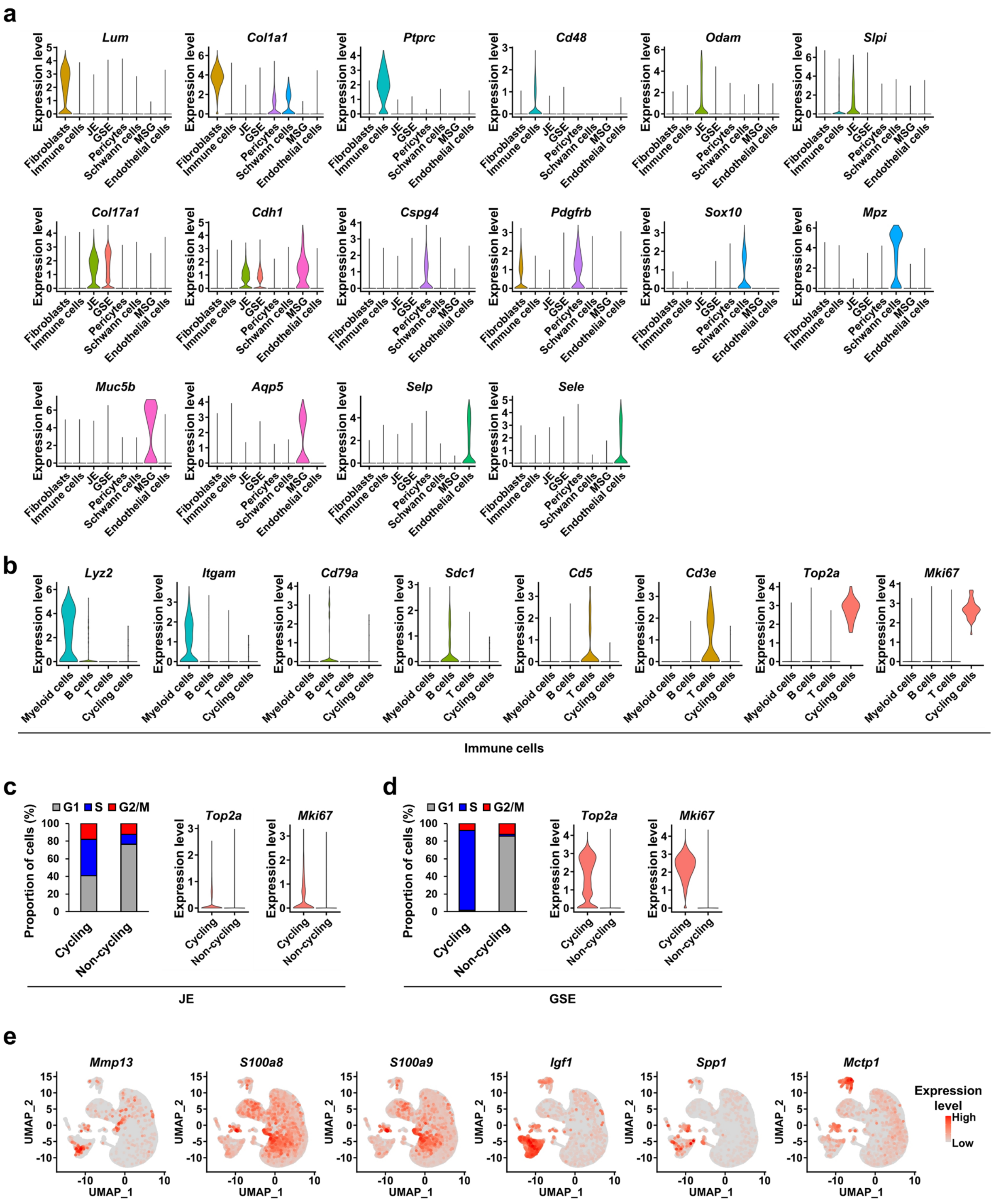

Supplementary Fig. 4: Marker genes used for cell-type clustering in mouse gingiva single-cell RNA-seq data.

a, b Violin plots indicating the expression of marker genes used to define cell types in total gingival cells (**a**) and immune cells (**b**).

c, d Stacked bar charts illustrating the proportion of cells in each cell cycle phase and the expression level of cell cycle-related genes across junctional epithelium (JE) (**c**) and gingival sulcular epithelium (GSE) (**d**) subpopulation.

e Feature plots showing the expression of six genes (*Mmp13, S100a8, S100a9, Igf1, Spp1,* and *Mctp1*) that are secreted and highly expressed in periodontitis-affected gingiva, alongside *Inhba.*
Source data are provided as a Source Data file.

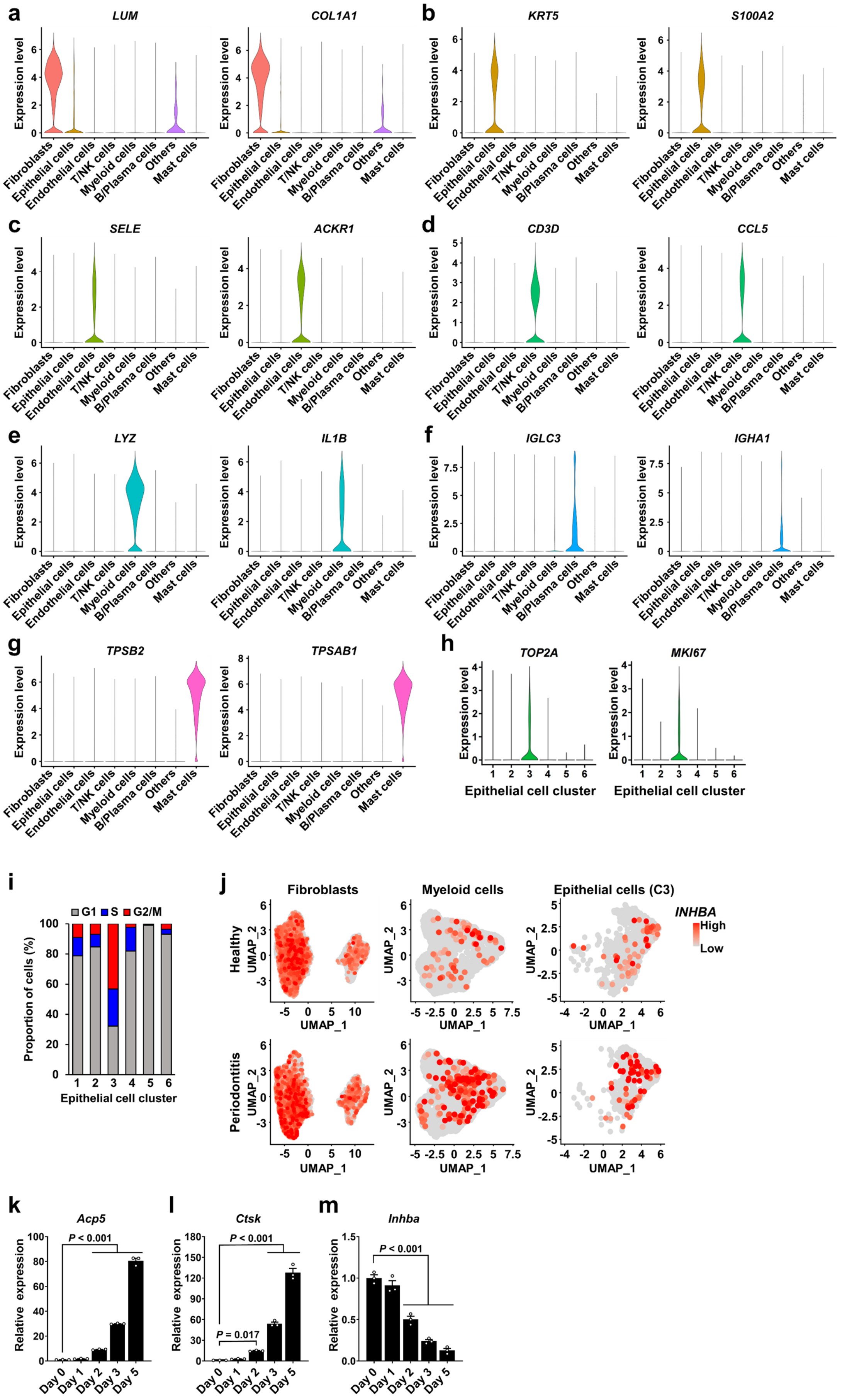
a
LUM
COL1A1
b
KRT5
S100A2
c
SELE
ACKR1
d
CD3D
CCL5
e
LYZ
IL1B
f
IGLC3
IGHA1
g
TPSB2
TPSAB1
h
TOP2A
MKI67
Expression level
Fibroblasts
Epithelial cells
Endothelial cells
T/NK cells
Myeloid cells
B/Plasma cells
Others
Mast cells
Epithelial cell cluster
i
G1 S G2/M
Proportion of cells (%)
Epithelial cell cluster
j
Fibroblasts
Myeloid cells
Epithelial cells (C3)
INHBA
High
Low
Healthy
Periodontitis
UMAP_1
UMAP_2
k
Acp5
l
Ctsk
m
Inhba
Relative expression
P < 0.001
P = 0.017
Day 0
Day 1
Day 2
Day 3
Day 5

Supplementary Fig. 5: Marker genes used for cell-type clustering in human gingiva single-cell RNA-seq data, and gene expression during osteoclastogenesis.

a-g Violin plots indicating the expression of marker genes used to define fibroblasts (**a**), epithelial cells (**b**), endothelial cells (**c**), T/NK cells (**d**), myeloid cells (**e**), B/Plasma cells (**f**), and mast cells (**g**).
h Expression of cell proliferation marker genes across epithelial cell clusters.
i Stacked bar chart illustrating the proportion of cells in each cell cycle phase across human epithelial cell clusters. Cluster 3, which has the highest overall *INHBA* expression, shows the greatest cell proliferation activity.
j Feature plots of *INHBA* expression in fibroblasts, myeloid cells, and epithelial cell (C3) in healthy and periodontitis-affected human gingiva.
k, l, m Expression of osteoclastogenesis marker genes, *Acp5* (**k**) and *Ctsk* (**l**), and *Inhba* (**m**) during osteoclast differentiation (n = 3 each).

Data (**k-m**) represent mean ± SEM. Statistical significance was assessed by one-way ANOVA with Holm-Šídák post hoc test (**k-m**). In (**k-m**), means of each time point were compared against the Day 0 group. Source data and exact P values are provided as a Source Data file.

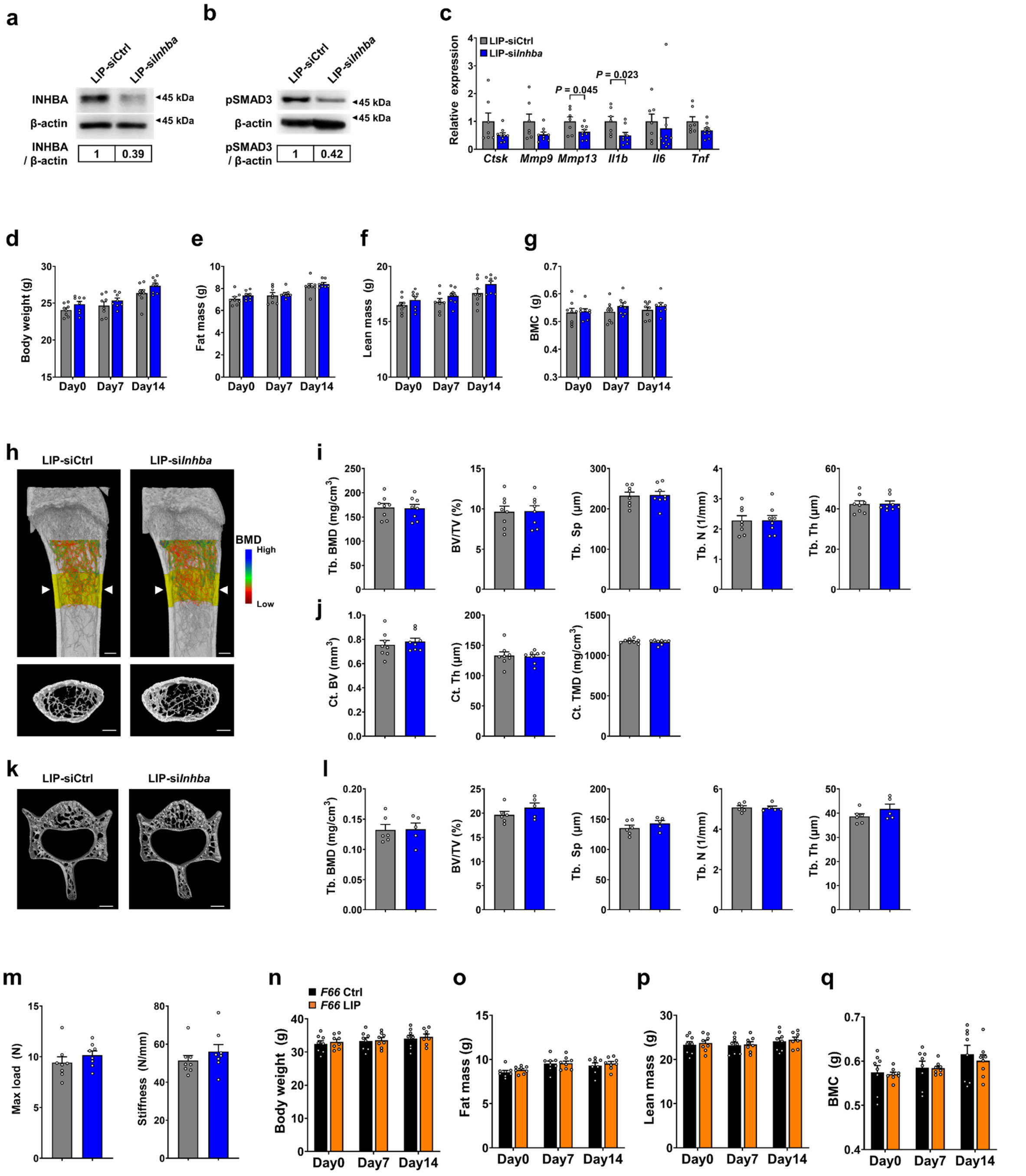

Supplementary Fig. 6: Effects of blocking activin A production and signaling.

a Representative Western blot of INHBA protein in gingival tissue from mice injected with siCtrl or si*Inhba* on day 7 after ligature placement.

b Representative Western blot of pSMAD3 in quadriceps muscles from mice injected with siCtrl or si*Inhba* on day 7 after ligature placement (*n* = 3 each).

c qRT-PCR expression analysis of genes related to tissue destruction (*Ctsk*, *Mmp9*, and *Mmp13*) and inflammation (*Il1b*, *Il6*, and *Tnf*), in the gingiva of mice injected with siCtrl or si*Inhba* on day 7 after ligature placement (LIP-siCtrl: *n* = 7, LIP-si*Inhba*: *n* = 9).

d, e, f, g Changes in body weight (**d**), fat mass (**e**), lean mass (**f**), and bone mineral content (BMC) (**g**)

after si*Inhba* injection (*n* = 8 each).

h, i, j Representative micro-CT images (**h**) and histomorphometric analyses of trabecular (**i**) and cortical (**j**) bone in the distal femur of mice injected with siCtrl or si*Inhba* 14 days after ligature placement (*n* = 8 each). BMD is indicated by color. The yellow region in the top panel indicates the area analyzed for cortical bone. The transverse view images of the area marked by arrowheads are displayed in the bottom panel. Scale bar, 0.5 mm.

k, l Representative micro-CT images (**k**) and histomorphometric analysis (**l**) of the lumbar spine in mice injected with siCtrl or si*Inhba* 14 days after ligature placement (LIP-siCtrl: *n* = 6, LIP-si*Inhba*: *n* = 5). Scale bar, 0.5 mm.

m Mechanical properties of the femur in mice injected with siCtrl or si*Inhba* on day 14 after ligature placement (*n* = 8 each).

n, o, p, q Changes in body weight (**n**), fat mass (**o**), lean mass (**p**), and BMC (**q**) after periodontitis induction in follistatin (*F66*) transgenic mice (*n* = 8 each).

Tb, Trabecular; BV/TV, Bone Volume/Total Volume; Tb. Sp, Tb. separation; Tb.N, Tb. number; Tb. Th, Tb. thickness; Ct. TMD, Ct. tissue mineral density. Data represent mean ± SEM. Statistical significance was assessed by two-tailed Student's *t*-test. Source data are provided as a Source Data file.

Supplementary Table 1: Association between periodontitis and grip strength in older adults (KNHANES analysis).

	Grip strength (kg) (Minimally adjusted)			Grip strength (kg) (Fully adjusted)		
Exposure	Mean (± SE)	β coefficients (95% CI)	*P* value	Mean (± SE)	β coefficients (95% CI)	*P* value
Periodontitis						
No	30.20 (± 0.19)	Reference		29.35 (± 0.50)	Reference	
Yes	29.67 (± 0.19)	-0.534 (-1.049 to -0.019)	0.042	28.81 (± 0.51)	-0.536 (-1.065 to -0.007)	0.047

Grip strength was adjusted for age and sex (Model 1, minimally adjusted) and additionally for body mass index, income, education level, diabetes, smoking status, and frequency of muscle-strengthening exercise (Model 2, fully adjusted), using complex-sample generalized linear model framework. The β-coefficients represent the mean difference in grip strength (kg) between the periodontitis and control groups. All tests were two-sided. KNHANES, Korea National Health and Nutrition Examination Survey; CI, confidence interval.

Supplementary Table 2: Demographic and clinical characteristics of the KNHANES study participants, stratified by periodontitis status.

	No periodontitis (*n* = 1144)	Periodontitis (*n* = 1017)	*P* value
Age, years	64.21 (± 2.82)	64.32 (± 2.89)	0.427
Sex			5.5E-09
Female	719 (63.9%)	488 (48.4%)	
Male	425 (36.1%)	529 (51.6%)	
Body mass index, kg/m^2	24.15 (± 2.94)	24.59 (± 3.17)	0.002
Income			6.4E-04
Q1 (lowest)	204 (17.8%)	222 (22.0%)	
Q2	207 (17.4%)	229 (23.4%)	
Q3	239 (20.5%)	202 (19.1%)	
Q4	233 (21.6%)	195 (19.7%)	
Q5 (highest)	257 (22.8%)	164 (15.8%)	
Education			0.032
Elementary or lower	396 (35.3%)	400 (41.2%)	
Middle school	226 (20.2%)	206 (21.6%)	
High school	288 (26.1%)	214 (21.3%)	
College or higher	192 (18.4%)	146 (15.9%)	
Diagnosed diabetes			0.055
Yes	174 (14.9%)	191 (18.3%)	
No	970 (85.1%)	826 (81.7%)	
Lifetime smoking (total)			4.0E-12
Never	784 (70.0%)	530 (52.6%)	
< 5 packs	12 (1.3%)	8 (0.9%)	
≥ 5 packs	337 (28.7%)	470 (46.5%)	
Muscle strengthening exercise, days/week	1.87 (± 1.68)	1.88 (± 1.72)	0.906

Values are presented as mean ± SD or n (%). Percentages for categorical variables were calculated based on participants with non-missing responses for each variable. Analyses were restricted to participants aged 60-69 years and accounted for the complex survey design and sampling weights. *P* values were obtained using complex-sample general linear models for continuous variables and Rao-Scott adjusted chi-square tests for categorical variables. All tests were two-sided. KNHANES, Korea National Health and Nutrition Examination Survey.

Supplementary Table 3: Characteristics of participants who provided serum samples, stratified by periodontitis status.

	Healthy (n = 12)	Periodontitis (n = 11)	P value
Age, years	54.50 (± 12.54)	62.73 (± 7.11)	0.067
Sex			0.414
Female	8 (66.7%)	5 (45.5%)	
Male	4 (33.3%)	6 (54.5%)	

Values are presented as mean ± SD or n (%). Periodontitis patients with alveolar bone loss extending to the mid-third of the root and beyond were included in the analysis. *P* values were calculated using Welch's *t*-test for continuous variables (age) and Fisher's exact test for categorical variables (sex). All tests were two-sided.

Supplementary Table 4: Characteristics of participants subjected to bulk RNA-sequencing analysis, stratified by periodontitis status.

	No periodontitis (*n* = 10)	Periodontitis (*n* = 10)	*P* value
Age, years	65.9 (± 7.37)	61.4 (± 6.09)	0.086
Sex			1.000
Female	5 (50%)	5 (50%)	
Male	5 (50%)	5 (50%)	

Values are presented as mean ± SD or n (%). Participants were excluded if they had a history of systemic conditions known to affect periodontal status, including uncontrolled diabetes mellitus, or if they were pregnant or breastfeeding at the time of enrollment. *P* values were calculated using Welch's *t*-test for continuous variables (age) and Fisher's exact test for categorical variables (sex). All tests were two-sided.

Supplementary Table 5: Characteristics of participants subjected to single-cell RNA sequencing analysis, stratified by periodontitis status.

	No periodontitis (*n* = 15)	Periodontitis (*n* = 9)	*P* value
Age band	21-65	34-65	
Sex			0.403
Female	10 (66.7%)	4 (44.4%)	
Male	5 (33.3%)	5 (55.6%)	
Race			0.157
African American	1 (6.7%)	0 (0%)	
Asian	5 (33.3%)	0 (0%)	
American Indian	1 (6.7%)	0 (0%)	
Caucasian	5 (33.3%)	4 (44.4%)	
Latinx	1 (6.7%)	3 (33.3%)	
Multiracial	0 (0%)	1 (11.1%)	
Not reported	2 (13.3%)	1 (11.1%)	

Age is reported as range due to unavailability of exact ages for all participants. Values are presented as n (%) for sex and race. *P* values were calculated using Fisher's exact test for sex and Fisher-Freeman-Halton exact test with Monte Carlo estimation for race. All tests were two-sided.

Supplementary Table 6: Primer sequences for qRT-PCR.

Gene	Primer sequence (5’-3’)
Inhba	F: GATCATCACCTTTGCCGAGT
	R: TGGTCCTGGTTCTGTTAGCC
Inhbb	F: CAGCTTTGCAGAGACAGATGG
	R: GTCTCCGTGACCCTGTTCTT
Inha	F: CGTACACCCTCCCAGTTTCA
	R: CAAAAACAGGGGCTGAACCG
Ctsk	F: GAAGAAGACTCACCAGAAGCAG
	R: TCCAGGTTATGGGCAGAGATT
Mmp9	F: GTCCAGACCAAGGGTACAGC
	R: ATACAGCGGGTACATGAGCG
Mmp13	F: GACAAGCAGTTCCAAAGGCTAC
	R: ATGGGAAACATCAGGGCTCC
Il1b	F: AGGAGAACCAAGCAACGACA
	R: CTTGGGATCCACACTCTCCAG
Il6	F: GTGGAAATGAGAAAAGAGTTGTGC
	R: TCCAGTTTGGTAGCATCCATCA
Tnf	F: CTTCTCATTCCTGCTTGTGGC
	R: ACTGATGAGAGGGAGGCCAT
Foxo3	F: TACGAGTGGATGGTGCGCTGT
	R: TCATTCTGAACGCGCATGAAGC
Fbxo32	F: TGAGCGACCTCAGCAGTTAC
	R: GCGCTCCTTCGTACTTCCTT
Trim63	F: TGCAGAGTGACCAAGGAGAATAG
	R: TTCTCGTCCAGGATGGCGTA
Acp5	F: CACTCCCACCCTGAGATTTGT
	R: CATCGTCTGCACGGTTCTG
18S	F: CTGCCCTATCAACTTTCGATGGTAG
	R: CCGTTTCTCAGGCTCCCTCTC

Uncropped scans of blots.

Supplementary Fig. 3a

INHBA

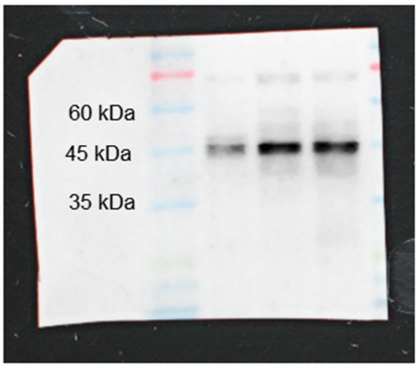

β-actin

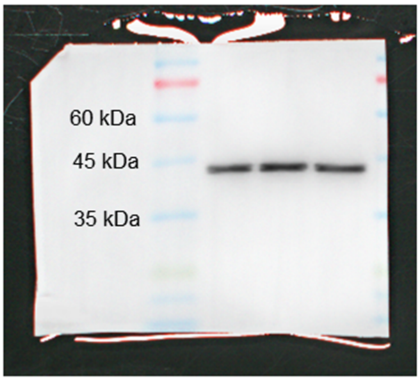

Supplementary Fig. 3b

pSMAD3

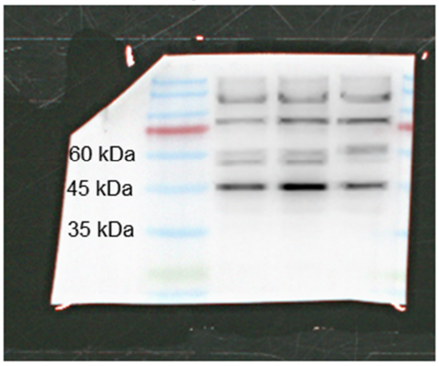

β-actin

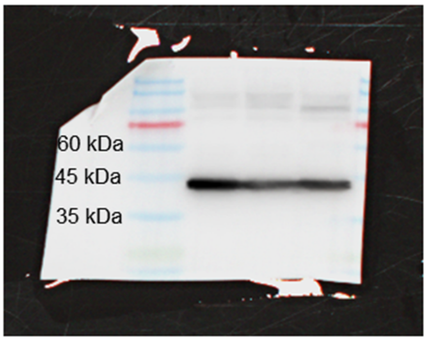

Supplementary Fig. 3d

MuRF1

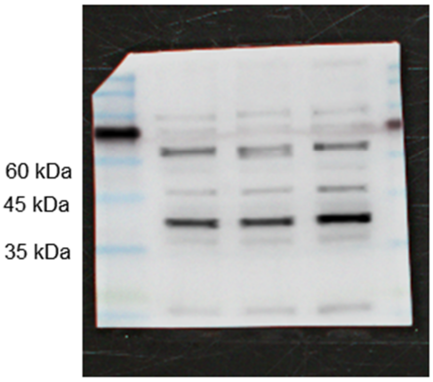

β-actin

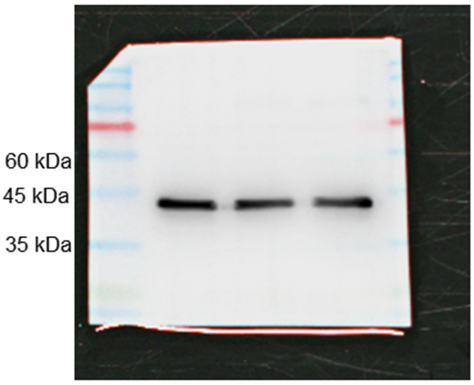

Supplementary Fig. 3j

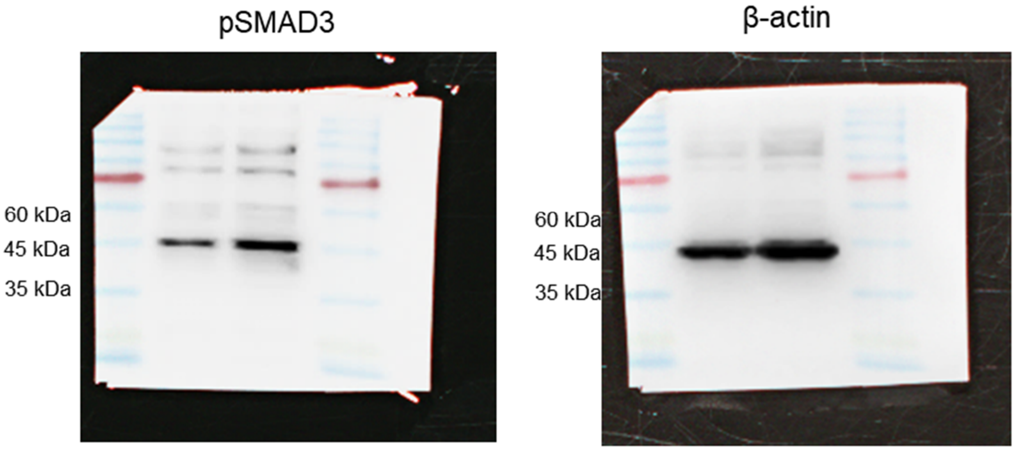

Supplementary Fig. 3l

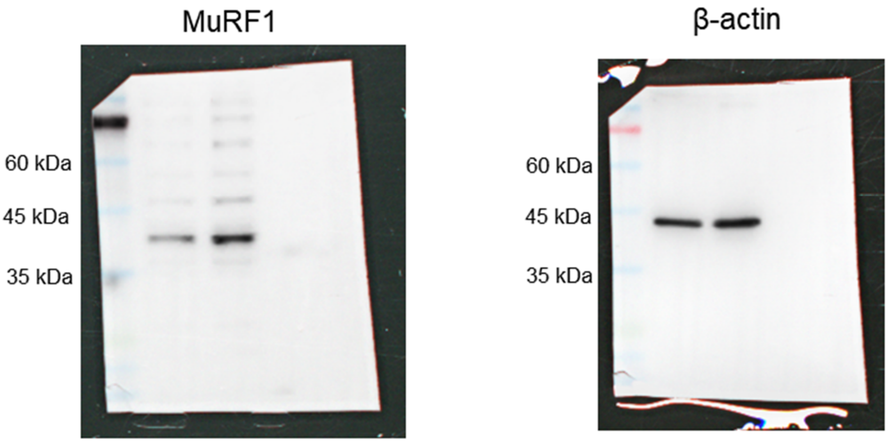

Supplementary Fig. 3m

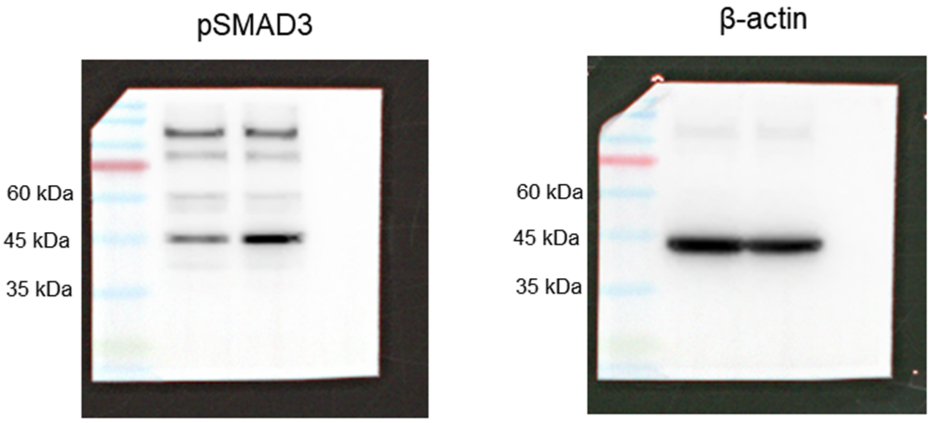

Supplementary Fig. 3o

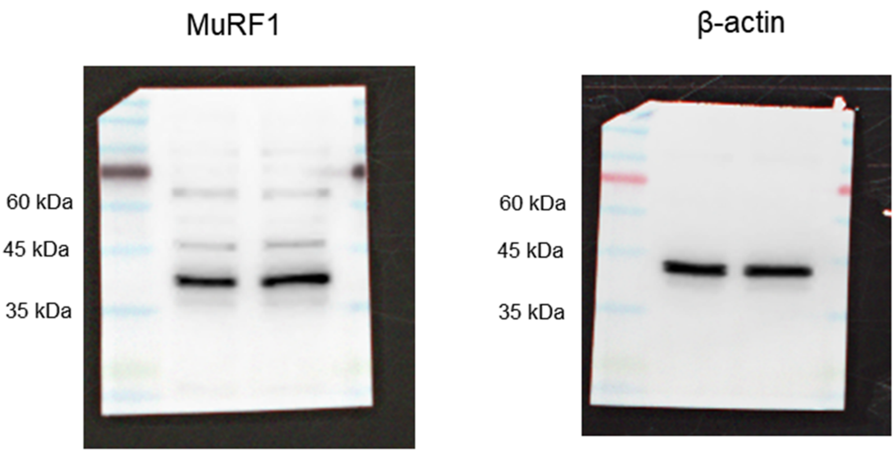

Supplementary Fig. 6a

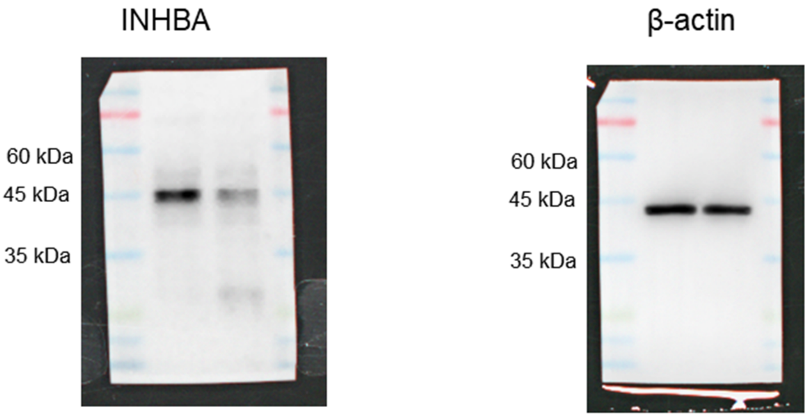

Supplementary Fig. 6b

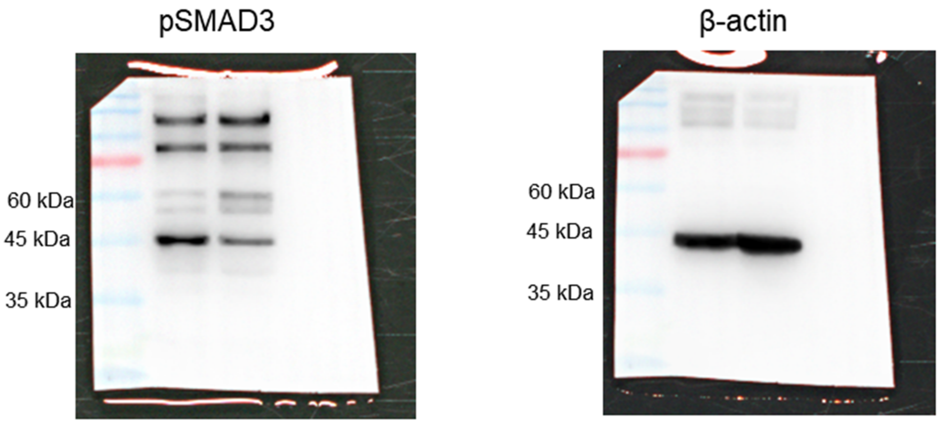

Supplement: Supplementary file 1 — Supplementary Information_resubmit [file 41467_2026_72766_MOESM1_ESM.pdf]
