# Supplementary material for: Periodontitis induces skeletal muscle atrophy by increasing circulating levels of activin A
Source: Nat Commun. 2026 May 6;17:4063. doi: 10.1038/s41467-026-72766-1 (PMC13149552; doi:10.1038/s41467-026-72766-1)
Supplement: Supplementary file 2 — Description of Additional Supplementary Files [file 41467_2026_72766_MOESM2_ESM.pdf]

Description of Additional Supplementary Files

Title: Supplementary Data 1

Description: Probe sequence for *Inhba* RNA fluorescence in situ hybridization.
