# Supplementary material for: Periodontitis induces skeletal muscle atrophy by increasing circulating levels of activin A
Source: Nat Commun. 2026 May 6;17:4063. doi: 10.1038/s41467-026-72766-1 (PMC13149552; doi:10.1038/s41467-026-72766-1)
Supplement: Supplementary file 3 — Dataset 1 [file 41467_2026_72766_MOESM3_ESM.pdf]

Supplementary Data 1: Probe sequence for *Inhba* RNA fluorescence in situ hybridization

5’-AGACACGTGATTTGCTTTTGCTCTTGAAGATGTGAACAGCATCTATGCATTCATTATCTCTGAC
CCCTCCAATAGCTTCTCAGTGATACAGGGTTTAATTTAAACACATACAATGTCCATCCCCCAACCT
CCTGCCCACATCTACAAGTCACACTTATGCGGGTTTTGGGGTGATTACATTTTTCTCGATACTAAA
AAGAGAAGTTGCCAAAAGGCCCACAGGAAATCATATTTTTCAAGTGACACAATCATTTGAATGCAT
GTTACTATAACTGAGGCATGTTTGTTCTGGAATCACAAAGTCACGTCTTGCGTTCAGTCTGGCAT
AGCTCCCAATTTTACTTATTCTGCCTTTTAAGAAAACACAAGGCCTCTCCTTGAAAATGGAGTGTG
GAATCAATCTTAAATGAACATAGAATTGGCTGGTCTTCTAAGATAAGAAAATCCCCATTGCTGTGC
TGAAATTTGACTTATTTTGGGAAGGAAAAGTGTCAATGAAGCTTACAAGTATTAAAGAGATACTAA
CTACAAATGCTCTTCAGAGCATCTAGAACTTGTGTGGGCAGGTATTTAAGAATTATCCTAGAGAAA
GTGGGGAGACCAGACAGAGCCACTTTAACACTAGGGAAAGCACACCATGAAGAAACTAAGATCT
TAAGCATTAAAGGCTTTTCAAATTTTGGATTAAGCACTTATGTAGAAAGACCAGTTCACAAGACTT
CATGTTTTCTAAATTGTTAACAATATCTAATATTGTTAAAATATTAGCAATTTAAATTCTATTGCAAG
GCCATAC-3’
