# Supplementary material for: Periodontitis induces skeletal muscle atrophy by increasing circulating levels of activin A
Source: Nat Commun. 2026 May 6;17:4063. doi: 10.1038/s41467-026-72766-1 (PMC13149552; doi:10.1038/s41467-026-72766-1)
Supplement: Supplementary file 4 — Reporting Summary [file 41467_2026_72766_MOESM4_ESM.pdf]

nature portfolio

Corresponding author(s): Yun-Sil Lee
Last updated by author(s): Mar 31, 2026

Reporting Summary

Nature Portfolio wishes to improve the reproducibility of the work that we publish. This form provides structure for consistency and transparency in reporting. For further information on Nature Portfolio policies, see our Editorial Policies and the Editorial Policy Checklist.

Statistics

For all statistical analyses, confirm that the following items are present in the figure legend, table legend, main text, or Methods section.

n/a	Confirmed	
☐	☒	The exact sample size (*n*) for each experimental group/condition, given as a discrete number and unit of measurement
☐	☒	A statement on whether measurements were taken from distinct samples or whether the same sample was measured repeatedly
☐	☒	The statistical test(s) used AND whether they are one- or two-sided<br>*Only common tests should be described solely by name; describe more complex techniques in the Methods section.*
☐	☒	A description of all covariates tested
☐	☒	A description of any assumptions or corrections, such as tests of normality and adjustment for multiple comparisons
☐	☒	A full description of the statistical parameters including central tendency (e.g. means) or other basic estimates (e.g. regression coefficient) AND variation (e.g. standard deviation) or associated estimates of uncertainty (e.g. confidence intervals)
☐	☒	For null hypothesis testing, the test statistic (e.g. *F*, *t*, *r*) with confidence intervals, effect sizes, degrees of freedom and *P* value noted<br>*Give P values as exact values whenever suitable.*
☒	☐	For Bayesian analysis, information on the choice of priors and Markov chain Monte Carlo settings
☒	☐	For hierarchical and complex designs, identification of the appropriate level for tests and full reporting of outcomes
☒	☐	Estimates of effect sizes (e.g. Cohen's *d*, Pearson's *r*), indicating how they were calculated

Our web collection on statistics for biologists contains articles on many of the points above.

Software and code

Policy information about availability of computer code

Data collection	Skyscan1272 (Bruker-MicroCT, ver.1.4), InAlyzer (Medikors), Zeiss LSM800 with Airyscan (Zeiss), Zeiss LatticeSIM5 (Zeiss), QuantStudio3Real-TimePCRSystem, SpectraMaxABS (MolecularDevices), Evolution-Capt Edge (Vilber), Bluehill Universal (Instron), AU480 Chemistry Analyzer (Beckman Coulter), Spark 10M (Tecan)
Data analysis	Excel (ver.16.0), GraphPad Prism (ver.10), CTAn (Bruker-MicroCT, ver.1.18), CTVox (Bruker-MicroCT, ver.3,3), DataViewer (Bruker-MicroCT, ver.1.5), InAlyzer (Medikors), ZEISS ZEN 3.10 (Zeiss), ImageJ, Cell Ranger (10X Genomics, ver.9.0.1), R (ver.4.1.2), edgeR (ver.3.36), QuantStudio Design & Analysis Software (Thermo Fisher Scientific, ver.1.5), STAR (ver.2.7.9a), Seurat (ver.5.1), DESeq (ver.1.34), Evolution-Capt Edge (Vilber), Bluehill Universal (Instron), SPSS (IBM, ver.29.0.1)

For manuscripts utilizing custom algorithms or software that are central to the research but not yet described in published literature, software must be made available to editors and reviewers. We strongly encourage code deposition in a community repository (e.g. GitHub). See the Nature Portfolio guidelines for submitting code & software for further information.

Data

Policy information about availability of data

All manuscripts must include a data availability statement. This statement should provide the following information, where applicable:

- Accession codes, unique identifiers, or web links for publicly available datasets
- A description of any restrictions on data availability
- For clinical datasets or third party data, please ensure that the statement adheres to our policy

The single-cell RNA-seq dataset generated in this study has been deposited in the GEO database under accession number GSE315263.
Previously generated RNA sequencing datasets are available in the GEO database under the accession codes: GSE152042, GSE164241, GSE186882, GSE223924.
The remaining data are available within the article, Supplementary information or Source data file. Source data are provided with this paper.

Research involving human participants, their data, or biological material

Policy information about studies with human participants or human data. See also policy information about sex, gender (identity/presentation), and sexual orientation and race, ethnicity and racism.

Reporting on sex and gender	Sex were matched between the groups. Sex was determined based on self-reporting.
Reporting on race, ethnicity, or other socially relevant groupings	Sex, education level, and income were self-reported by participants and were included as covariates to control for potential confounding in the association between periodontitis and grip strength. To address the potential confounding, we used complex-samples generalized linear models.
Population characteristics	Age were matched between the groups.
Recruitment	The biospecimens and data used for this study were provided by the Biobank of Seoul National University Dental Hospital, a member of the Korea Biobank Network (project No. 2024ER050701).
Ethics oversight	Institutional Review Board of Seoul National University

Note that full information on the approval of the study protocol must also be provided in the manuscript.

Field-specific reporting

Please select the one below that is the best fit for your research. If you are not sure, read the appropriate sections before making your selection.

☒ Life sciences ☐ Behavioural & social sciences ☐ Ecological, evolutionary & environmental sciences

For a reference copy of the document with all sections, see nature.com/documents/nr-reporting-summary-flat.pdf

Life sciences study design

All studies must disclose on these points even when the disclosure is negative.

Sample size	The sample size for each experiment is represented by individual dots in the figures, and specified in the figure legends. The sample size were determined using RNASeqPower or based on previously published studies in this field.
Data exclusions	Data were not excluded from analysis.
Replication	At least three replicates were performed. All attempts at replication were successful.
Randomization	Mice were randomly assigned to different experimental groups.
Blinding	Investigator blinding was not possible during the experiments, as the ligature used to induce periodontitis is visually apparent, making it impossible to conceal group assignment from the experimenter.

Reporting for specific materials, systems and methods

We require information from authors about some types of materials, experimental systems and methods used in many studies. Here, indicate whether each material, system or method listed is relevant to your study. If you are not sure if a list item applies to your research, read the appropriate section before selecting a response.

Materials & experimental systems

n/a	Involved in the study	
☐	☒	Antibodies
☒	☐	Eukaryotic cell lines
☒	☐	Palaeontology and archaeology
☐	☒	Animals and other organisms
☐	☒	Clinical data
☒	☐	Dual use research of concern
☒	☐	Plants

Methods

n/a	Involved in the study	
☒	☐	ChIP-seq
☒	☐	Flow cytometry
☒	☐	MRI-based neuroimaging

Antibodies

Antibodies used

anti-DIG antibody (1:500, Roche, 11333089001)
anti-Ki-67 antibody (1:1,000, Abcam, ab15580)
anti-CD11b antibody (1:100, Invitrogen, 14-0112-82)
anti-FLAG antibody (1:400, Cell Signaling Technology, D6W5B)
anti-ACVR2A antibody (1:15, R&D Systems, AF340)
anti-ACVR2B antibody (1:15, R&D Systems, AF339)
anti-GFP antibody (1:1,250, Cell Signaling Technology, 2555)
anti-MHC type I antibody (1:100, DSHB, BA-D5)
anti-MHC type IIa antibody (1:100, DSHB, SC-71)
anti-MHC type IIb antibody (1:50, DSHB, BF-F3)
anti-INHBA antibody (1:1,000, Abcam, ab128958)
anti-phospho-SMAD3 antibody (1:1,000, Cell Signaling Technology, 9520)
anti-MuRF1 antibody (1:1,000, Santa Cruz, sc-32920)
anti-β-actin(C4)-HRP (1:1,000, Santa Cruz, sc-47778 HRP)
Donkey anti-sheep Alexa 488 secondary antibody (1:200, Invitrogen, A-11015)
Goat anti-rabbit Alexa 488 secondary antibody (1:500, Invitrogen, A-11008)
Goat anti-rabbit Alexa 594 secondary antibody (1:400, Invitrogen, A-11012)
Goat anti-rat Alexa 488 secondary antibody (1:500, Invitrogen, A-11007)
Donkey anti-goat Alexa Plus 488 secondary antibody (1:500, Invitrogen, A-32814)
Goat anti-mouse Alexa Fluor 594 secondary antibody (1:200, Invitrogen, A-11032)
Goat anti-mouse Alexa Fluor 488 secondary antibody (1:200, Invitrogen, A-21042)
Goat anti-rabbit HRP (1:4,000, Invitrogen, 31460)

Validation

anti-DIG antibody (Roche, 11333089001); PMID:34848703
anti-Ki-67 antibody (Abcam, ab15580); https://www.abcam.com/en-us/products/primary-antibodies/ki67-antibody-ab15580
anti-CD11b antibody (Invitrogen, 14-0112-82); https://www.thermofisher.com/antibody/product/CD11b-Antibody-clone-M1-70-Monoclonal/14-0112-82
anti-FLAG antibody (Cell Signaling Technology, D6W5B); https://www.cellsignal.com/products/primary-antibodies/dykddddk-tag-d6w5b-rabbit-monoclonal-antibody-binds-to-same-epitope-as-sigma-aldrich-anti-flag-m2-antibody/14793
anti-ACVR2A antibody (R&D Systems, AF340); https://www.rndsystems.com/products/human-activin-riia-antibody_af340
anti-ACVR2B antibody (R&D Systems, AF339); https://www.rndsystems.com/products/human-activin-riib-antibody_af339
anti-GFP antibody (Cell Signaling Technology, 2555); https://www.cellsignal.com/products/primary-antibodies/gfp-antibody/2555
anti-MHC type I antibody (DSHB, BA-D5); https://dshb.biology.uiowa.edu/BA-D5
anti-MHC type IIa antibody (DSHB, SC-71); https://dshb.biology.uiowa.edu/SC-71
anti-MHC type IIb antibody (DSHB, BF-F3); https://dshb.biology.uiowa.edu/BF-F3
anti-INHBA antibody (Abcam, ab128958); https://www.abcam.com/en-us/products/primary-antibodies/inhibin-beta-a-antibody-epr27852-ab128958
anti-phospho-SMAD3 antibody (Cell Signaling Technology, 9520); https://www.cellsignal.com/products/primary-antibodies/phospho-smad3-ser423-425-c25a9-rabbit-monoclonal-antibody/9520
anti-MuRF1 antibody (Santa Cruz, sc-32920); PMID: 22476919
anti-β-actin(C4)-HRP (Santa Cruz, sc-47778 HRP); https://www.scbt.com/ko/p/beta-actin-antibody-c4

Animals and other research organisms

Policy information about studies involving animals; ARRIVE guidelines recommended for reporting animal research, and Sex and Gender in Research

Laboratory animals

Eight-week-old C57BL/6 mice and eight-week-old F66 mice on a C57BL/6 background were used. Mice were maintained under a 12-hour light/dark cycle at an ambient temperature of 23°C and 50% relative humidity.

Wild animals

The study did not involve wild animals.

Reporting on sex

All animal experiments were conducted using male mice only to minimize physiological variability associated with the estrous cycle. Furthermore, in the F66 transgenic line used in this study, the follistatin transgene is integrated into the Y chromosome, resulting in overexpression exclusively in males.

Field-collected samples	The study did not involve samples collected from the field.
Ethics oversight	All animal studies were approved by the Institutional Animal Care and Use Committee policies (Seoul National University). (SNU-220914-4-1, SNU-230410-4-5, SNU-260102-1)

Note that full information on the approval of the study protocol must also be provided in the manuscript.

Clinical data

Policy information about clinical studies

All manuscripts should comply with the ICMJE guidelines for publication of clinical research and a completed CONSORT checklist must be included with all submissions.

Clinical trial registration	The study did not involve a clinical trial.
Study protocol	Serum samples were obtained from periodontally healthy individuals and patients with periodontitis. Periodontitis status and severity were determined based on clinical diagnosis and panoramic radiographs, with radiographic staging assigned according to the 2017 World Workshop on the Classification of Periodontal and Peri-Implant Diseases and Conditions.
Data collection	Serum samples were obtained from individuals who visited Seoul National University Dental Hospital between January 1, 2021 and June 30, 2025 and provided informed consent for biobanking.
Outcomes	Serum activin A levels were measured using Quantikine Activin A ELISA Kit (R&D Systems) following the manufacturer's instructions and compared between periodontally healthy individuals and patients with periodontitis.

Plants

Seed stocks	Not applicable
Novel plant genotypes	Not applicable
Authentication	Not applicable
